# Sex-related disparities in migraine recognition and management: insights from a tertiary headache center cohort

**DOI:** 10.1186/s10194-025-02189-8

**Published:** 2025-10-20

**Authors:** Cornelius Angerhöfer, Carolin Luisa Hoehne, Marlene Ulrich, Kristin Sophie Lange, Mira Pauline Fitzek, Yones Salim, Uwe Reuter, Lucas Hendrik Overeem, Bianca Raffaelli

**Affiliations:** 1 Department of Neurology with Experimental Neurology, Charité-Universitätsmedizin Berlin, Freie Universität Berlin and Humboldt-Universität zu Berlin, Charitéplatz 1, 10117 Berlin, Germany; 2 Clinician Scientist Program, Berlin Institute of Health (BIH), Berlin, Germany; 3 Universitätsmedizin Greifswald, Greifswald, Germany

**Keywords:** Sex differences, Migraine recognition, Clinical presentation, Diagnostic accuracy, Treatment recommendations

## Abstract

**Background:**

Migraine is a highly prevalent and disabling neurological disorder that is often underdiagnosed and undertreated, particularly in early stages of care. Sex-related factors may contribute to this gap. This study investigates sex-related disparities in migraine recognition and management before and after presentation at a tertiary headache center and examines whether these disparities are linked to differences in clinical presentation.

**Methods:**

In this retrospective cross-sectional study, we included patients diagnosed with migraine between December 2015 and January 2023 at the Headache Center of Charité – Universitätsmedizin Berlin. We analyzed sex-related differences in prior diagnosis, clinical presentation, migraine burden, and treatment strategies both before referral and following tertiary care evaluation.

**Results:**

Among 1,130 patients with migraine (82% women, 18% men), men were significantly less likely than women to have received a migraine diagnosis prior to tertiary center evaluation (57.0% vs. 73.8%; OR = 0.47, *p* < 0.001). Clinical presentation also differed between sexes: men reported lower pain intensity and shorter attack duration, whereas women more frequently experienced unilateral headache, nausea, vomiting, photophobia, and osmophobia. In women, canonical migraine features (unilateral headache, pulsating pain, aggravation by physical activity, and migraine-associated symptoms) were each associated with lower odds of missed diagnosis before referral. By contrast, in men, pressing headache quality substantially increased the likelihood of missed diagnosis. Women were also more likely than men to have used triptans and received prophylactic treatment prior to tertiary evaluation.

**Conclusions:**

Men with migraine are substantially less likely than women to receive an accurate diagnosis in routine care. While canonical migraine features facilitate recognition in women, non-prototypical symptoms reduce diagnostic accuracy in men. These findings reveal a critical gap in clinical awareness that may contribute to persistent underdiagnosis and delayed treatment in men.

**Clinical trial number:**

Not applicable.

**Graphical Abstract:**

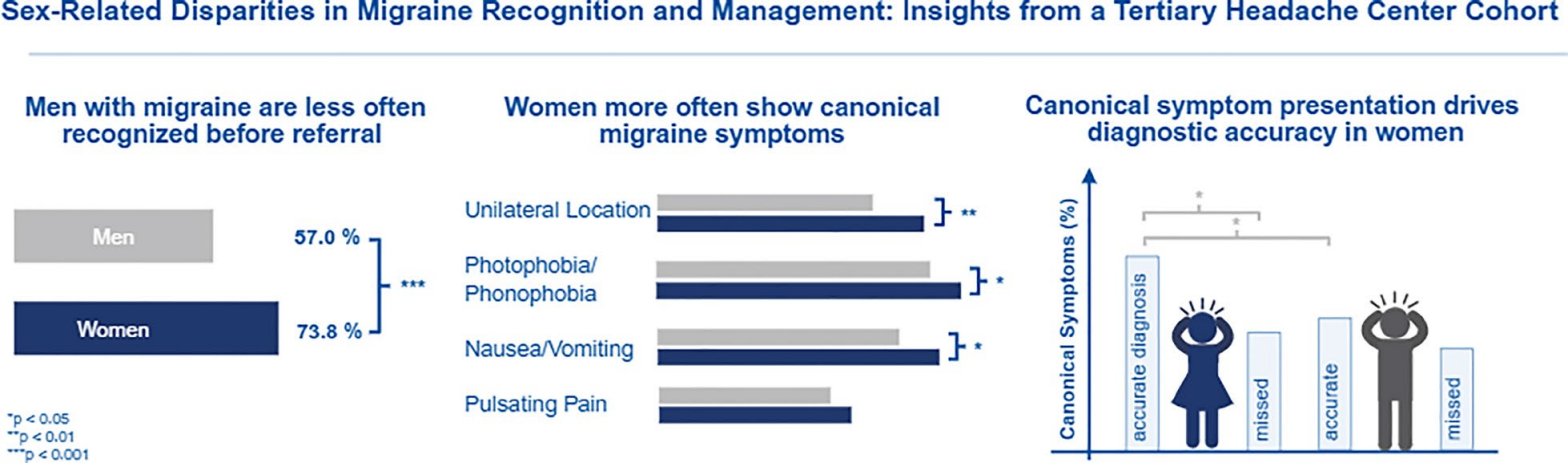

**Supplementary Information:**

The online version contains supplementary material available at 10.1186/s10194-025-02189-8.

## Background

Migraine is one of the most prevalent neurological disorders worldwide, affecting approximately 15% of the global population [1]. It ranks as the leading cause of years lived with disability among individuals under the age of 50 [2], significantly diminishing quality of life and overall well-being. Despite its high prevalence and substantial impact, migraine is still frequently under- or misdiagnosed, often leading to significant delays in reaching an accurate diagnosis and effective treatment [3, 4]. Contributing factors include limited consultation time during early-stage care and the clinical overlap between migraine and other headache disorders [5, 6]. Our recent findings underscore this challenge: nearly one-third of patients received their first migraine diagnosis only after referral to a specialized tertiary headache center [7], while the majority of patients meeting criteria for prophylactic treatment were not receiving any such therapy in primary or secondary care [8].

Sex-related biases may further compound challenges in migraine recognition and treatment. The prevalence of migraine is two- to three times higher in women than in men [9], which has mistakenly contributed to the misconception and clinical stigma of migraine as a “women’s disorder” [10]. Moreover, clinical presentations differ by sex. Men with migraine tend to report fewer hallmark symptoms such as unilateral, pulsating pain, aggravation by routine activity as well as migraine-associated symptoms including photophobia, phonophobia, nausea, vomiting, and osmophobia [11–15]. Some studies have also reported that women experience more intense pain and longer-lasting attacks as well as higher attack frequency [12, 15–18]. Whether these differences reflect biological factors or are instead influenced by cultural expectations and self-stigma, which arise from the perception that migraine is a “women’s disorder” and that men should not express pain, remains a subject of debate. However, these findings have not been consistently supported across the literature [19, 20].

Regarding sex differences in migraine treatment, studies have reported similar response rates for triptans, although women experience higher rates of side effects and rebound headache [21]. Preventive treatment with calcitonin gene-related peptide (CGRP) monoclonal antibodies appears to show no sex-related differences in efficacy [22, 23]. By contrast, CGRP receptor antagonists (gepants) for acute treatment seem to be more effective in women than in men [24]. Interestingly, a recent large-scale online survey revealed that men are considerably more likely to use acute treatments, yet less likely to engage in prophylactic therapies [18]. Despite these findings, sex differences in the use of acute treatments and commonly prescribed preventive medications remain largely understudied.

To gain a deeper understanding of sex-related disparities in migraine care, this study examines differences in diagnosis and treatment between men and women. We examine these disparities both before and after presentation at our tertiary headache center and explore whether they are linked to differences in clinical presentation.

## Methods

### Study design & participants

This is a post-hoc analysis of a retrospective cross-sectional cohort study among patients who presented for their initial consultation at the headache center at Charité – Universitätsmedizin Berlin between December 2015 and January 2023. For detailed methods, please refer to the primary publication [7].

All patients were referred from either the primary care level (i.e., general practitioner) or the secondary care level (i.e., neurologist or pain medicine specialist). Before their first visit to the tertiary headache center, patients were invited to complete a comprehensive self-reported medical history questionnaire. Variables collected from the questionnaire included birth year, sex, height, weight, duration of the disease, and the presence of any previous headache or non-headache diagnosis. Moreover, patients were asked to fill out patient-reported outcome measurements (PROM). The PROMs included three questionnaires, each available in a validated German version. The Headache Impact Test-6 (HIT-6) assesses headache severity and its impact on daily functioning [25]. The Depression, Anxiety and Stress Scale-21 (DASS-21) categorizes the severity of depression, anxiety, and stress symptoms [26]. The Short Form-12 (SF-12) provides physical and mental health composite scores, with lower scores indicating a greater disease burden [27].

At their first visit to our tertiary center, patients were evaluated by an attending physician who established a diagnosis regardless of any previous assessments from other healthcare providers. A standardized report (doctor’s letter) was then prepared, summarizing the clinical findings, diagnosis, and treatment recommendations. The attending physician was either a board-certified neurologist with specialized training in headache medicine or a neurology resident under close supervision. From these standardized reports, we extracted detailed clinical data, including confirmed or suspected headache diagnosis, headache frequency, and onset. Additionally, comprehensive information on clinical presentation was gathered, encompassing headache location, presence of migraine aura, pain characteristics, attack duration, and headache intensity measured on a 1-to-10 visual analog scale (VAS), as well as migraine-associated symptoms. Descriptors of pain quality were grouped into two categories: pressing/dull (hereafter referred to as ‘pressing’) or pulsating/throbbing (hereafter referred to as ‘pulsating’). Data on prior treatments and current acute and preventive treatment recommendations were also recorded.

We included all patients who attended their initial visit during the study period, completed the self-reported medical history questionnaire, and received a migraine diagnosis documented in the standardized report. Classification into definite or probable migraine was retrospectively confirmed based on the International Classification of Headache Disorders, 3rd edition (ICHD-3) criteria for migraine with or without aura [7, 28]. Definite migraine cases fulfilled all required criteria, while probable cases met all but one.

### Objective

The primary objective of this study was to examine sex-related differences in migraine diagnosis prior to presentation at our tertiary headache center and to identify headache characteristics that may contribute to these variations. Additionally, we aimed to explore sex-related differences in prior acute and preventive treatment strategies, as well as in treatment recommendations provided at the tertiary care level.

### Statistical analysis

All analyses were conducted in R (R Foundation for Statistical Computing, Vienna, Austria) within the RStudio v2024.12.1 + 563 environment (Posit PBC, Boston, MA, USA). The sample size was determined by data availability rather than formal power calculation. Continuous variables (e.g., age, BMI, disease duration, headache characteristics) were summarized as mean ± standard deviation (SD) and compared between sexes using the Mann–Whitney U test. Mean differences with 95% confidence intervals (CIs) were calculated assuming independent samples. Binary variables (diagnosis, comorbidities, headache characteristics, treatments) were reported as counts and percentages, with sex-specific proportions compared using chi-square tests of independence. For each 2 × 2 comparison, absolute risk differences with 95% CIs were estimated. Ordinal treatment variables (e.g., acute therapies) were collapsed into four categories (0, 1, 2, ≥ 3) and compared across sexes using chi-square tests.

To examine association between diagnostic accuracy prior to tertiary center presentation and clinical characteristics, participants were classified into four diagnostic groups: women with (prior) accurate diagnosis, men with (prior) accurate diagnosis, women with missed diagnosis, and men with missed diagnosis. To examine sex-specific predictors of missed migraine diagnosis prior to tertiary center presentation, logistic regression models were fitted with the binary outcome (missed diagnosis) as the dependent variable. Each predictor was entered as an interaction term with sex (sex × predictor). Odds ratios (ORs) with 95% Wald CIs were reported, along with interaction p-values. Multiple testing across variable sets was addressed using false discovery rate (FDR) adjustment via the Benjamini–Hochberg procedure. All p-values were two-sided, with *p* < 0.05 considered statistically significant. Analyses were performed using complete-case data without imputation. The study follows the Strengthening the Reporting of Observational Studies in Epidemiology guidelines for reporting observational cohort studies [29].

## Results

### Patient selection & characteristics

Between December 2015 and January 2023, a total of 3,264 patients attended their first consultation at our tertiary headache center and were given out comprehensive history questionnaires. Of 1,780 returned, 364 were excluded (280 due to missing medical records, 32 unrelated to headache, 52 lacking diagnostic information), leaving 1,416 evaluable cases. Of these, 1,168 patients had been diagnosed with definite or probable migraine according to ICHD-3 criteria. An additional 38 were excluded due to missing diagnostic information, leaving 1,130 patients for inclusion in the primary analysis.

The final cohort comprised 930 women and 200 men, with a median age of 42.3 years (Table 1). Age, age at migraine onset and disease duration did not differ by sex. Men had a higher BMI (*p* < 0.001). An additional headache diagnosis was made in 220 patients, with tension-type headache more frequent in men than in women (13.0% vs. 7.1%, *p* = 0.020; Table 1).

**Table 1:** Study population

	**Mean ± SD / n (%)**			**Effect size**	***p*** **-values**	
**Demographic characteristics**	***Men (n = 200)***	***Women (n = 930)***	**Total** ***(N = 1130)***	**Mean difference (95% CI)**	***p***	***p*** ** (FDR BH)**
Age	41.6 ± 14.2	42.5 ± 13.3	42.3 ± 13.4	-0.82 (-2.96–1.32)	0.348	0.465
BMI	23.8 ± 8.8	22.8 ± 8.4	30.0 ± 8.5	0.99 (-0.35–2.32)	< 0.001***	< 0.001***
Age at Onset	21.3 ± 14.1	20.5 ± 12.2	20.7 ± 12.6	0.80 (-1.31–2.90	0.890	0.890
Disease Duration	19.8 ± 15.4	21.7 ± 14.4	21.4 ± 14.6	-1.91 (-4.23–0.42)	0.037*	0.075
**Additional diagnosis**	**Men**	**Women**	**Total**	**Indifference difference (95% CI)**	***p***	***p*** ** (FDR BH)**
Tension-Type Headache	26 (13.0%)	66 (7.1%)	92 (8.1%)	5.9% (1.0% to 10.8%)	0.006**	0.020*
Cluster Headache	0 (0%)	2 (0.2%)	2 (0.2%)	-0.2% (-0.5% to 0.1%)	0.512	0.598
Other	21 (10.5%)	113 (12.2%)	134 (11.9%)	-1.7% (-6.4% to 3.1%)	0.512	0.598

*p < 0.05, **p < 0.01, ***p < 0.001

### Comorbidities

Overall, the most common comorbidities were sleep disorders (36.2%), bruxism (30.6%), depression (22.8%), anxiety (18.8%), and thyroid diseases (19.3%) (Supplementary Table 1). Thyroid diseases, including both hypo- and hyperthyroidism, were significantly more common in women (22.4% vs. 5.0%, *p* < 0.001). There was a trend for hypertension to be more frequent in men (19.5% vs. 12.4%, *p* = 0.053), while bruxism tended to be more common in women (31.9% vs. 24.5%, *p* = 0.180); however, neither difference reached statistical significance.

### Migraine recognition

Before referral to our headache center, men were significantly less likely than women to have received an accurate migraine diagnosis (57.0% vs. 73.8%, *p* < 0.001). Logistic regression analysis confirmed this difference, showing that men had markedly lower odds of prior accurate diagnosis (OR = 0.47, 95% CI 0.34–0.66, *p* < 0.001). Accordingly, men were more than twice as likely as women to present to the tertiary center without a prior migraine diagnosis (OR = 2.12, 95% CI 1.52–2.94, *p* < 0.001).

### Clinical characteristics

Significant sex-related differences were observed in the clinical presentation of migraine (Table 2). Compared with women, men reported significant lower average pain intensity on the VAS (7.00 vs. 7.34, *p* = 0.014) and shorter attack durations, both for minimum (24.3 h vs. 42.3 h, *p* < 0.001) and maximum estimates (42.6 h vs. 60.4 h, *p* < 0.001). The number of monthly headache days did not differ between sexes. With respect to pain characteristics, women significantly more often reported unilateral headache location (75.9% vs. 65.0%, *p* = 0.007) and moderate to severe pain intensity (99.1% vs. 97.0%, *p* = 0.027). In contrast, no sex-related differences were observed for aggravation by physical activity or for pain quality. Women reported migraine-associated symptoms significantly more frequent than men, including vomiting (38.8% vs. 29.0%, *p* = 0.023), nausea (81.8% vs. 71.0%, *p* = 0.005), photophobia (86.5% vs. 80.0%, *p* = 0.039) and osmophobia (28.4% vs. 18.2%, *p* = 0.013). A trend toward higher rates of phonophobia in women was not significant (84.1% vs. 79.5%, *p* = 0.164).

**Table 2:** Headache and pain characteristics

	***Mean ± SD / n ( %)***			**Effect Size**	***p*** **-values**	
**Headache characteristics**	**Men**	**Women**	**Total**	**Mean difference (95% CI)**	***P***	***p*** ** (FDR BH)**
Pain Intensity (VAS)	7.00 ± 1.46	7.34 ± 1.50	7.28 ± 1.50	-0.35 (-0.57 to -0.12)	0.010*	0.014*
Minimum Duration (h)	24.3 ± 54.0	42.3 ± 80.7	39.1 ± 77.0	-18.0 (-27.5 to -8.4)	< 0.001***	< 0.001***
Maximum Duration (h)	42.6 ± 72.8	60.4 ± 80.9	57.3 ± 79.8	-17.6 (-29.7 to -6.0)	< 0.001***	< 0.001***
Mean Number of Monthly Headache Days	12.8 ± 8.7	12.3 ± 7.5	12.4 ± 7.7	0.48 (-0.8–1.8)	0.957	0.957
**Pain characteristics**				**Incidence difference (95% CI)**		
Unilateral Location	130 (65.0%)	706 (75.9%)	836 (74.0%)	-10.9% (-18.1% to -3.8%)	0.001**	0.007**
Pain Intensity (moderate/severe)	191 (97.0%)	901 (99.1%)	1092 (98.7%)	-2.2% (-4.6% to 0.3%)	0.014*	0.027*
Pulsating	101 (50.5%)	509 (54.7%)	610 (54.0%)	-4.2% (-11.9% to 3.4%)	0.276	0.301
Pressing	142 (71.0%)	615 (66.1%)	757 (67.0%)	4.9% (-2.1% to 11.9%)	0.184	0.230
Aggravation by Physical Activity	168 (93.3%)	774 (93.8%)	942 (93.7%)	-0.5% (-4.5% to 3.5%)	0.808	0,808
**Migraine-associated symptoms**						
Vomiting	58 (29.0%)	361 (38.8%)	419 (37.1%)	-9.8% (-16.8% to -2.8%)	0.009**	0.023*
Nausea	142 (71.0%)	761 (81.8%)	903 (79.9%)	-10.8% (-17.6% to -4.1%)	< 0.001***	0.005**
Photophobia	160 (80.0%)	804 (86.5%)	964 (85.3%)	-6.5% (-12.4% to -0.5%)	0.019*	0.039*
Osmophobia	34 (18.2%)	258 (28.4%)	292 (26.7%)	-10.3% (-16.5% to -4.0%)	0.004**	0.013*
Phonophobia	159 (79.5%)	782 (84.1%)	941 (83.3%)	-4.6% (-10.7% to 1.5%)	0.115	0.164

*p < 0.05, **p < 0.01, ***p < 0.001

### Migraine symptoms by diagnostic group

**Fig. 1:**
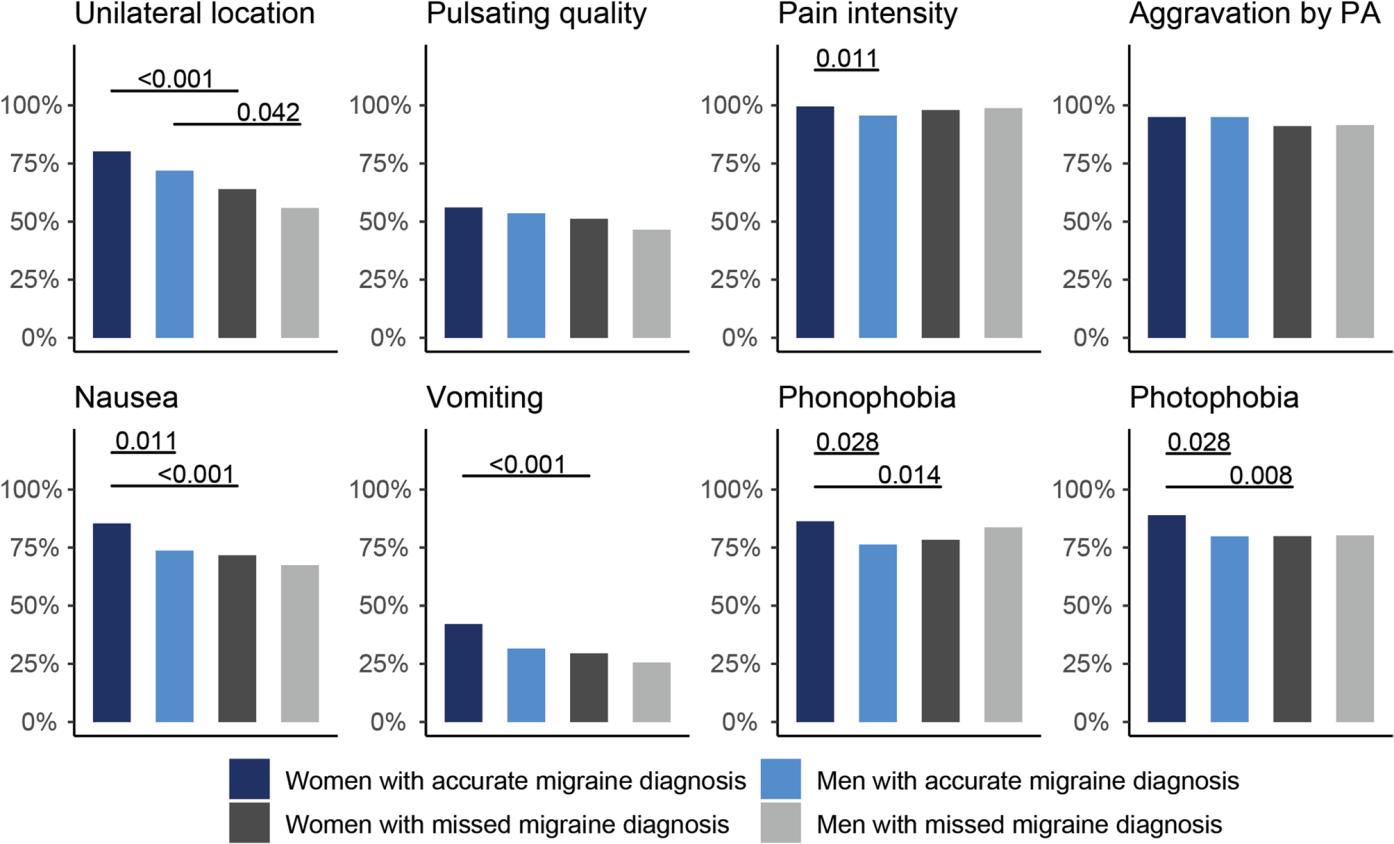
Sex-specific characteristics, stratified by diagnostic group. Bar plots show the proportion of men and women with unilateral location, pulsating quality, pain intensity (moderate/severe), aggravation by physical activity, nausea, vomiting, phonophobia and photophobia, stratified by accurate versus missed migraine diagnosis. Dark blue = women with accurate diagnosis, light blue = men with accurate diagnosis, dark gray = women with missed diagnosis, light gray = men with missed diagnosis. FDR-adjusted p-values are displayed above each comparison. PA, Physical Activity

Unilateral pain location was significantly more frequent in both women and men with an accurate diagnosis prior to tertiary center presentation compared to those with a missed diagnosis (women: 80.2% vs. 63.9%, *p* < 0.001; men: 63.9% vs. 55.8%, p *=* 0.042; Fig. 1). Women with an accurate diagnosis also reported significantly higher rates of migraine-associated symptoms (nausea, vomiting, phonophobia, and photophobia) than women with a missed diagnosis and, with the exception of vomiting, than men with an accurate diagnosis (all p *<* 0.05, Fig. 1). Furthermore, moderate to severe pain intensity was more frequently reported by women with an accurate diagnosis than by men with an accurate diagnosis (99.6% vs. 95.6%, p *=* 0.011). By contrast, no significant sex-related differences were observed between accurate and missed diagnoses regarding aggravation by physical activity or pulsating pain.

### Sex-symptom interaction in migraine recognition

**Fig. 2:**
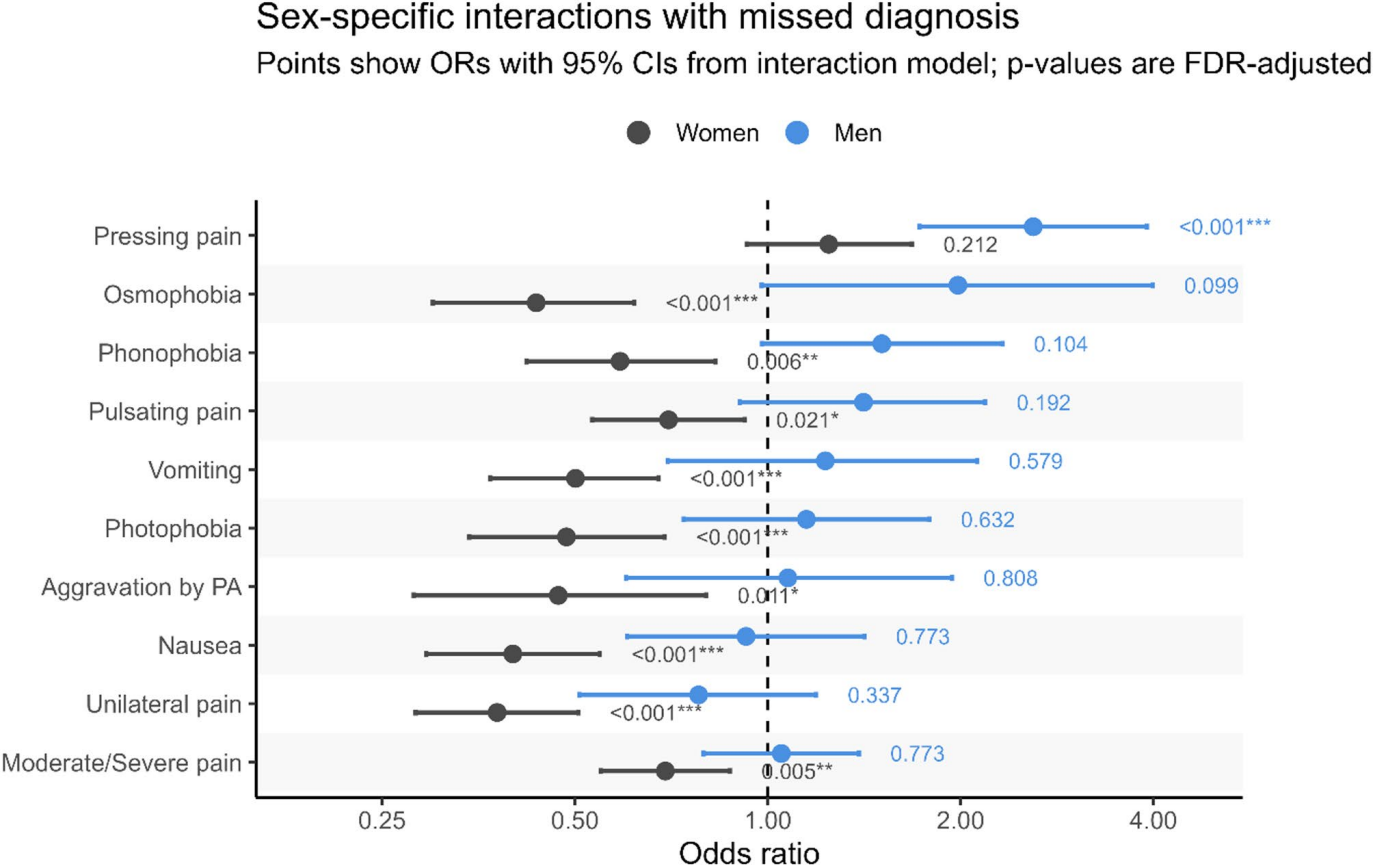
Sex-specific interactions with missed migraine diagnosis. Forest plot showing odds ratios (ORs, log scale) and 95% confidence intervals (CIs) for the association between symptoms and the likelihood of missed diagnosis. Separate estimates are presented for women (gray) and men (blue). The dashed vertical line indicates the null value (OR = 1). Labels to the right of each confidence interval show FDR-adjusted p-values for the sex-specific interaction terms. PA, Physical Activity. *p < 0.05, **p < 0.01, ***p < 0.001

Migraine recognition depended not only on headache characteristics, but also their interaction with patient sex (Fig. 2). In women, unilateral pain location (OR = 0.38, *p* < 0.001), moderate to severe pain intensity (OR = 0.69, *p* = 0.005), aggravation by physical activity (OR = 0.47, *p* = 0.011), and pulsating pain (OR = 0.70, *p* = 0.021) were all associated with significantly lower odds of missed migraine diagnosis prior to tertiary presentation, a pattern not seen in men. Similarly, migraine-associated symptoms including osmophobia (OR = 0.44, *p* < 0.001), phonophobia (OR = 0.59, *p* = 0.006), photophobia (OR = 0.49, *p* < 0.001), nausea (OR = 0.40, *p* < 0.001), and vomiting (OR = 0.50, *p* < 0.001) were linked to reduced odds of missed migraine diagnose in women, but showed no significant association in men. In contrast, among men, pressing headache quality (OR = 2.60, *p* < 0.001) was strongly associated with increased odds of missed migraine diagnosis before tertiary evaluation.

### Patient-reported outcome measurements

Significant sex differences were observed in Patient-Reported Outcome Measures (PROMs) (Table 3). Women had a significantly higher HIT-6 score compared to men (56.4 ± 6.2 vs. 54.7 ± 6.0, *p* = 0.002), indicating greater headache impact. In contrast, DASS-21 subscores for depression, stress, and anxiety showed no significant differences between sexes. Men reported a significantly lower physical burden than women, as reflected by a higher score on the SF-12 physical component (40.1 ± 10.2 vs. 38.1 ± 9.4, p *=* 0.034), while the mental component showed no sex-related differences.

**Table 3:** Patient-related outcome measurements (PROMs)

	Mean ± SD			Effect Size	*p*-values	
PROMs	Male	Female	Total	Mean difference (95% CI)	*p*	*p* (FDR BH)
HIT-6	54.7 ± 6.0	56.4 ± 6.2	56.1 ± 6.2	-1.72 (-2.66 to -0.79)	< 0.001***	0.002**
DASS-21 (Depression)	6.4 ± 5.5	6.0 ± 4.9	6.1 ± 5.0	0.37 (-0.46–1.20)	0.681	0.681
DASS-21 (Stress)	7.6 ± 4.9	8.1 ± 4.6	8.0 ± 4.7	-0.48 (-1.22–0.27)	0.120	0.150
DASS-21 (Angst)	3.6 ± 3.6	4.1 ± 3.7	4.0 ± 3.7	-0.43 (-0.99–0.13)	0.081	0.150
SF-12 Physical Component	40.1 ± 10.2	38.1 ± 9.4	38.5 ± 9.5	1.94 (0.35–3.53)	0.011*	0.034*
SF-12 Mental Component	42.3 ± 12.7	41.1 ± 11.1	41.3 ± 11.4	1.17 (-0.78–3.13)	0.125	0.150

*p < 0.05, **p < 0.01, ***p < 0.001

### Acute treatment

Overall, 69.6% of patients had used a triptan for acute migraine treatment prior to presentation at the tertiary center (Table 4). Women were significantly more likely than men to have used one or more triptans before referral (72.5% vs. 56.5%, *p* < 0.001). Additionally, 77.9% of patients reported prior use of acute non-migraine-specific treatments (non-MST), including nonsteroidal anti-inflammatory drugs (NSAIDs), paracetamol (acetaminophen) and combination analgesics containing caffeine or other agents. No significant sex differences were observed in the use of non-MSTs before tertiary center presentation.

**Table 4:** Prior use of triptans and non–migraine specific treatments (non-MST)

	Counts			Effect Size	*p*-values	
Acute treatment	Male *n* (%)	Female *n* (%)	Total *n* (%)	Incidence difference (95% CI)	*p*	*p* (FDR BH)
Prior Triptan use	113 (56.5%)	674 (72.5%)	787 (69.6%)	-16.0% (-23.4% to -8.5%)	< 0.001***	< 0.001***
*None*	87 (43.5%)	256 (27.5%)	343 (30.4%)			
*1 triptan*	82 (41%)	400 (43%)	482 (42.7%)			
*2 triptans*	26 (13%)	181 (19.5%)	207 (18.3%)			
*≥ 3 triptans*	5 (2.5%)	93 (10%)	98 (8.7%)			
Prior non-MST	159 (79.5%)	721 (77.5%)	880 (77.9%)	2.0% (-4.2% to 8.2%)	0.542	0.542
*None*	41 (20.5%)	209 (22.5%)	250 (22.1%)			
*1 non-MST*	86 (43%)	379 (40.8%)	365 (32.3%)			
*2 non-MST*	40 (20%)	209 (22.5%)	249 (22.0%)			
*≥ 3 non-MST*	33 (16.5%)	133 (14.3%)	166 (14.7%)			

non-MST = non–migraine-specific treatment

*p < 0.05, **p < 0.01, ***p < 0.001

### Prophylactic treatment

Prior to presenting at our headache center, women had significantly more often received a prophylactic treatment compared to men (47.2% vs. 38.0%, *p* = 0.044; Table 5). Regarding specific drug classes, women tended to have used antiepileptic drugs (30.2% vs. 21.0%, *p* = 0.284) and beta-blockers (28.6% vs. 21.0%, *p* = 0.456) more frequently than men. However, none of these differences reached statistical significance (Table 5). Following tertiary center consultation, women were significantly more often advised to initiate a new treatment regimen compared to men (64.3% vs. 54.0%, *p* = 0.038), while no sex-related differences were observed in recommendations for initiating specific drug classes (Table 6). Women were also more frequently advised to discontinue their current treatment (3.5% vs. 0.5%, *p* = 0.044), whereas recommendations to continue or adjust existing therapies did not differ between sexes (Table 6).

**Table 5:** Prophylactic treatment prior to tertiary center presentation

	Counts			Effect Size	*p*-values	
Prophylactic treatment	Male *n*(%)	Female *n*(%)	Total *n*(%)	Incidence difference, 95% CI	*p*	*p* (FDR BH)
Prior prophylactic treatment	76 (38%)	439 (47.2%)	515 (45.6%)	-9.2% (-16.7% to -1.8%)	0.018*	0.044*
Mean number of prior prophylactic treatments	0.71 ± 1.24	1.06 ± 1.47	1.00 ± 1.44	-0.35 (-0.54 to -0.15)	0.001**	0.001**
* Antiepileptics*	42 (21%)	281 (30.2%)	323 (28.6%)	-9.2% (-15.6% to -2.8%)	0.009**	0,284
* Beta-Blocker*	42 (21%)	266 (28.6%)	308 (27.3%)	-7.6% (-14.0% to -1.3%)	0.028*	0,456
* Tricyclic Antidepressant*	44 (22%)	262 (28.2%)	306 (27.1%)	-6.2% (-12.6% to 0.3%)	0.075	0,478
* Calcium-Channel-Blocker*	15 (7.5%)	75 (8.1%)	90 (8%)	-0.6% (-4.6% to 3.5%)	0.789	0,876
* Onabotulinumtoxin-A*	6 (3%)	61 (6.6%)	67 (5.9%)	-3.6% (-6.4% to -0.7%)	0.053	0,478
* SSRIs / SNRIs*	5 (2.5%)	41 (4.4%)	46 (4.1%)	-1.9% (-4.4% to 0.6%)	0.215	0,574
* CGRP(R)-mAb*	1 (0.5%)	18 (1.9%)	19 (1.7%)	-1.4% (-2.8% to -0.1%)	0.152	0,554

CGRP(R)-mAb = Calcitonin Gene-Related Peptide (Receptor) - Monoclonal Antibody

SSRIs / SNRIs = Selective Serotonin Reuptake Inhibitors / Serotonin–Norepinephrine Reuptake Inhibitors

*p < 0.05, **p < 0.01, ***p < 0.001﻿

**Table 6:** Prophylactic treatment recommendations at the tertiary center

	Counts			Effect Size	*p*-values	
Recommendation	Male *n* (%)	Female *n* (%)	Total *n* (%)	Incidence difference, 95% CI	*p*	*p* (FDR BH)
Continue current treatment	9 (4.5%)	68 (7.3%)	77 (6.8%)	-2.8% (-6.1% to 0.5%)	0.152	0,183
Adjust current treatment	16 (8%)	70 (7.5%)	86 (7.6%)	0.5% (-3.7% to 4.6%)	0,819	0,819
Stop current treatment	1 (0.5%)	33 (3.5%)	34 (3%)	-3.0% (-4.6% to -1.5%)	0.022*	0.044*
Start with a new treatment	108 (54.0%)	598 (64.3%)	706 (62.5%)	-10.3% (-17.9% to -2.7%)	0.006**	0.038*
* Antiepileptics*	27 (13.5%)	161 (17.3%)	188 (16.6%)	-3.8% (-9.1% to 1.5%)	0.189	0,554
* Onabotulinumtoxin-A*	26 (13%)	143 (15.4%)	169 (15%)	-2.4% (-7.6% to 2.8%)	0.393	0,785
* Tricyclic Antidepressant*	29 (14.5%)	121 (13%)	150 (13.3%)	1.5% (-3.8% to 6.8%)	0,573	0,823
* Beta-Blocker*	21 (10.5%)	123 (13.2%)	144 (12.7%)	-2.7% (-7.5% to 2.0%)	0.294	0,673
* CGRP(R)-mAb*	8 (4%)	52 (5.6%)	60 (5.3%)	-1.6% (-4.7% to 1.5%)	0.363	0,773
* Calcium-Channel-Blocker*	4 (2%)	45 (4.8%)	49 (4.3%)	-2.8% (-5.2% to -0.5%)	0.074	0,478
* SSRIs / SNRIs*	7 (3.5%)	42 (4.5%)	49 (4.3%)	-1.0% (-3.9% to 1.9%)	0.522	0,823

CGRP(R)-mAb = Calcitonin Gene-Related Peptide (Receptor) - Monoclonal Antibody

SSRIs / SNRIs = Selective Serotonin Reuptake Inhibitors / Serotonin–Norepinephrine Reuptake Inhibitors

*p < 0.05, **p < 0.01, ***p < 0.001

## Discussion

In this large retrospective study among 1,130 patients with migraine, the rate of accurate migraine diagnosis before tertiary center evaluation was significantly lower in men compared to women. Only 57% of male participants had received a correct diagnosis prior to presentation at our tertiary headache center, compared to over 73% of female participants. These findings reflect a modest improvement over those reported by a previous German study [30], who found that only 41% of men meeting ICHD-3 criteria for migraine had received a corresponding diagnosis. However, direct comparison is limited by differences in study design, as their data were drawn from a population-based cohort. In our analysis, male sex was associated with a significantly lower odds of receiving an accurate migraine diagnosis (OR = 0.47). Compared to the cross-sectional study by Lipton et al. [14], in which men were over four times less likely than women to be correctly diagnosed, our findings may indicate a positive trend toward improved recognition of migraine in men. Nonetheless, differences in study populations constrain direct comparison, and our results underscore that underdiagnosis in men remains a significant and persistent issue.

The sex differences in migraine presentation observed in this study may help explain disparities in diagnostic recognition between men and women. Consistent with previous research [11, 12, 18, 21], our findings show that women report significantly more intense pain, longer attack duration, and more frequent unilateral pain and migraine-associated symptoms compared to men. Canonical migraine features were most common among women who received an accurate diagnosis prior to tertiary referral, suggesting that clinical presentations closely matching the typical migraine phenotype facilitate earlier and more accurate recognition, particularly in women. A significant sex–symptom interaction further supports this interpretation: in women, canonical migraine features (unilateral pain, moderate to severe intensity, pulsating pain, aggravation by physical activity, nausea, vomiting, photophobia, and phonophobia) were all associated with lower odds of missed diagnosis, whereas in men, non-prototypical features, such as pressing pain, were linked to a higher likelihood of diagnostic oversight. Overall, these findings suggest that non-prototypical symptom profiles may hinder diagnostic clarity in men, while canonical symptom presentations enhance diagnostic accuracy in women. Recognizing these sex-related patterns has important clinical implications. Increased clinician awareness of non-canonical migraine features in men, such as pressing or bilateral pain, and further re-evaluation of current diagnostic guidelines, may help reduce diagnostic delays and improve access to appropriate care.

Higher HIT-6 scores indicated a greater overall disease burden in women, consistent with previous reports [12, 17, 26], whereas no sex differences were observed in psychiatric symptoms such as depression, anxiety, and stress. Men reported lower physical impact, which may reflect reporting behavior rather than true differences, potentially influenced by stigma around acknowledging physical limitations in men [31].

Women were significantly more likely to use one or more triptans as acute medication compared to men, while we did not find any sex-related differences regarding non-specific acute treatment. This contrasts with a recent large Polish study reporting that men are generally more likely to use acute treatments, including both physician-prescribed (mostly triptans) and over-the-counter medications [18]. The discrepancy may reflect differences in study populations: their study was a cross-sectional online survey, whereas ours included only patients seeking tertiary care evaluation, introducing selection bias. In our cohort, women were also significantly more likely to have received prophylactic treatment prior to tertiary consultation. This finding aligns with the Polish study, which reported less frequent prophylactic use among men [18]. Notably, women in our cohort were more often advised to initiate a new prophylactic regimen following tertiary evaluation, potentially reflecting a higher likelihood of inadequate response to prior treatments. Despite methodological differences in patient selection between our prior report [8] and the present analysis, our findings remain consistent, supporting the robustness of our findings.

While the results highlight important sex-related disparities in the presentation of migraine and management, several limitations should be acknowledged. First, this was a retrospective cross-sectional study conducted at a single tertiary care center in Germany, which may limit the generalizability of the findings to other clinical settings or populations. The analysis was based on biological sex rather than gender identity, which limits the ability to capture the experiences of gender-diverse individuals and to fully address aspects of stigma. Moreover, the analysis did not account for life-stage differences, which may influence migraine presentation and diagnostic patterns. Future research should stratify by age or hormonal stage to better understand these dynamics. Also, it is important to emphasize that this present cohort consisted solely of patients referred to a tertiary headache center, representing a more severe and treatment-resistant subset of the overall migraine population. This referral bias may limit the generalizability of our findings to patients successfully managed in primary or secondary care. Accordingly, replication in larger, prospective studies across multiple international headache centers is necessary to validate and extend these observations. Second, the observational and cross-sectional design of the study precludes establishing causal relationships between sex and diagnostic or treatment disparities. Third, data on prior diagnosis and treatments relied on patient self-report, which is subject to recall bias and potential inaccuracies in reporting, potentially influencing the observed diagnostic and treatment patterns. Finally, although standardized clinical documentation protocols were used, some degree of variability in physician judgment and clinical decision-making is inevitable, which may have introduced subtle inconsistencies in patient assessment and management.

## Conclusions

This study reveals clear sex-related disparities in migraine recognition. Men were significantly less likely than women to receive an accurate migraine diagnosis, a difference attributed to less canonical symptom presentations despite a comparable overall migraine burden. These findings underscore the need to enhance recognition of non-prototypical migraine features, such as pressing pain, to reduce diagnostic delays and facilitate timely, effective management in men.

Additionally, examining migraine recognition and presentation across different life stages may reveal age-dependent patterns in symptom expression and diagnostic delay. Future prospective, multicenter studies involving diverse populations, also including gender-diverse individuals are necessary to confirm these patterns and to further explore the underlying factors driving sex-related disparities in migraine recognition and care.

## Supplementary Information

Below is the link to the electronic supplementary material.

Supplementary Material 1

## Data Availability

Data is provided within the manuscript or supplementary files.

## Abbreviations

CGRP: Calcitonin gene-related peptide
HIT-6: Headache Impact Test-6
DASS-21: Depression, Anxiety and Stress Scale-21
SF-12: Short Form-12
VAS: Visual analog scale
ICHD-3: International Classification of Headache Disorders, 3rd edition
non-MST: non–migraine-specific treatment
SSRIs: Selective serotonin reuptake inhibitors
SNRIs: Serotonin–norepinephrine reuptake inhibitors
