# Supplementary material for: Sex-related disparities in migraine recognition and management: insights from a tertiary headache center cohort
Source: J Headache Pain. 2025 Oct 20;26(1):225. doi: 10.1186/s10194-025-02189-8 (PMC12539007; doi:10.1186/s10194-025-02189-8)
Supplement: Supplementary file 1 — Supplementary Material 1 [file 10194_2025_2189_MOESM1_ESM.docx]

**Sex-Related Disparities in Migraine Recognition and Management: Insights from a Tertiary Headache Center Cohort**

**Supplementary Material**

Cornelius Angerhöfer*^1^, Carolin Luisa Hoehne*^1^, Marlene Ulrich^1^, Kristin Sophie Lange^1,2^, Mira Pauline Fitzek^1,2^, Yones Salim^1^, Uwe Reuter^1,3^, Lucas Hendrik Overeem*^1^, Bianca Raffaelli*^1,2^

*these authors contributed equally

Affiliation

^1^ Department of Neurology, Charité-Universitätsmedizin Berlin, corporate member of Freie Universität Berlin and Humboldt-Universität zu Berlin, Berlin, Germany

^2^ (Junior) Clinician Scientist Program, Berlin Institute of Health (BIH), Berlin, Germany

^3^ Universitätsmedizin Greifswald, Greifswald, Germany

Correspondence

Bianca Raffaelli (https://orcid.org/0000-0001-9758-1494)

Charité – Universitätsmedizin Berlin

Department of Neurology with Experimental Neurology

Charitéplatz 1

10117 Berlin, Germany

bianca.raffaelli@charite.de

**Table 1 Comorbidities**

|  | **Counts (%)** | | | **Effect Size** | **p-values** | |
| --- | --- | --- | --- | --- | --- | --- |
| **Comorbidity** | **Male** | **Female** | **Total** | **Incidence diff (95% CI)** | **p** | **p (FDR BH)** |
| Thyroid diseases | 10 (5%) | 208 (22.4%) | 218 (19.3%) | -17.4% (-21.4% to -13.3%) | < 0.001*** | < 0.001*** |
| Hypertension | 39 (19.5%) | 115 (12.4%) | 154 (13.6%) | 7.1% (1.2% to 13.0%) | 0.008** | 0.053 |
| Bruxism | 49 (24.5%) | 297 (31.9%) | 346 (30.6%) | -7.4% (-14.1% to -0.8%) | 0.038* | 0.180 |
| Gastrointestinal disease | 17 (8.5%) | 119 (12.8%) | 136 (12%) | -4.3% (-8.7% to 0.1%) | 0.090 | 0.316 |
| Anxiety | 34 (17%) | 178 (19.1%) | 212 (18.8%) | -2.1% (-7.9% to 3.6%) | 0.482 | 0.752 |
| Diabetes | 7 (3.5%) | 24 (2.6%) | 31 (2.7%) | 0.9% (-1.8% to 3.7%) | 0.470 | 0.752 |
| Heart disease | 13 (6.5%) | 52 (5.6%) | 65 (5.8%) | 0.9% (-2.8% to 4.6%) | 0.617 | 0.752 |
| Hyperlipidemia | 16 (8%) | 66 (7.1%) | 82 (7.3%) | 0.9% (-3.2% to 5.0%) | 0.655 | 0.752 |
| Kidney disease | 9 (4.5%) | 48 (5.2%) | 57 (5%) | -0.7% (-3.9% to 2.5%) | 0.698 | 0.752 |
| Liver disease | 9 (4.5%) | 32 (3.4%) | 41 (3.6%) | 1.1% (-2.0% to 4.2%) | 0.467 | 0.752 |
| Respiratory disease | 27 (13.5%) | 111 (11.9%) | 138 (12.2%) | 1.6% (-3.6% to 6.7%) | 0.540 | 0.752 |
| Sleep disorders | 67 (33.5%) | 342 (36.8%) | 409 (36.2%) | -3.3% (-10.5% to 4.0%) | 0.382 | 0.752 |
| Stroke | 2 (1%) | 13 (1.4%) | 15 (1.3%) | -0.4% (-2.0% to 1.2%) | 0.656 | 0.752 |
| Depression | 47 (23.5%) | 211 (22.7%) | 258 (22.8%) | 0.8% (-5.7% to 7.3%) | 0.804 | 0.804 |
